# IJCH Successfully Completes Transition Year

**DOI:** 10.3402/ijch.v71i0.20315

**Published:** 2012-12-28

**Authors:** Kue Young

As 2012 draws to a close, I am delighted to report to our readers that IJCH has successfully transformed itself into an Open Access and completely online journal, joining the family of journals published by the capable hands of our colleagues in Co-Action.

Without fixed publication dates, manuscripts are now published online as soon as they are copyedited and laid out, greatly reducing the time lapse between submission and publication.

The Thomson impact factor (IF) for 2011 has been released. Our IF is 1.060, a slight decline from 2010 (1.065). We ranked 78 out of 131 in the category of Public, Environmental and Occupational Health. Another competing IF is the SJR which uses different math and is based on Scopus data rather than the Web of Science. Our score is 0.049, which places us in the 2^nd^ highest quartile in the Medicine (Misc) category. Clearly, continuing improvement of the quality of the journal is our priority.

The year 2012 also witnessed important changes in the journal's parent organization, the International Association of Circumpolar Health Publishers (IACHP). It has merged with the International Network for Circumpolar Health Research (INCHR) to form a new, stronger organization called Circumpolar Health Research Network (CircHNet). The new network consists of both institutional and individual members, and in addition to publishing IJCH, is also engaged in educational activities and promotion of international collaboration in research.

The 15^th^ International Congress of Circumpolar Health was convened in August, 2012 in Fairbanks, Alaska. As with past congresses, it was a major success, which shall be evident when its proceedings are published in our journal as a Special Issue in 2013.

An important change in 2013 will be the implementation of a 500 euro publication fee for articles that have been accepted for publication. All manuscripts submitted prior to January 1, 2013 and already under review or production will be exempt from this fee. Individuals with financial difficulty can apply to Co-Action for a waiver. Ability to pay the fee will in no way affect or influence the editorial decision process. We consider this an important step towards making the journal financially self-sustaining. Our fees are quite low by the standards of most online Open Access journals.

We thank the former Editor Dr. Tiina Ikäheimo and assistant Riitta Aittamaa in our former editorial office in Oulu, Finland, for facilitating the transition and finalizing the closure of IACHP. The new board members of CircHNet have worked tirelessly to kick-start the new organization, under the leadership of our President, Professor Peter Bjerregaard of Copenhagen.

We look forward to a new and exciting year in 2013. Let me wish all our readers a Merry Christmas and a Happy New Year.

*Kue Young* Editor-in-Chief

